# Diet Quality and Sarcopenia in Older Adults: A Systematic Review

**DOI:** 10.3390/nu10030308

**Published:** 2018-03-05

**Authors:** Ilse Bloom, Calum Shand, Cyrus Cooper, Sian Robinson, Janis Baird

**Affiliations:** 1 MRC Lifecourse Epidemiology Unit, University of Southampton, Southampton General Hospital, Southampton SO16 6YD, UK; calum.shand@gmail.com (C.S.); cc@mrc.soton.ac.uk (C.C.); smr@mrc.soton.ac.uk (S.R.); jb@mrc.soton.ac.uk (J.B.); 2 NIHR Southampton Biomedical Research Centre, University of Southampton and University Hospital Southampton NHS Foundation Trust, Southampton SO16 6YD, UK; 3 NIHR Musculoskeletal Biomedical Research Unit, University of Oxford, Oxford OX3 7LE, UK

**Keywords:** ageing, diet quality, muscle, older people, physical function, sarcopenia

## Abstract

The increasing recognition of sarcopenia, the age-related loss of skeletal muscle mass and function (muscle strength and physical performance), as a determinant of poor health in older age, has emphasized the importance of understanding more about its aetiology to inform strategies both for preventing and treating this condition. There is growing interest in the effects of modifiable factors such as diet; some nutrients have been studied but less is known about the influence of overall diet quality on sarcopenia. We conducted a systematic review of the literature examining the relationship between diet quality and the individual components of sarcopenia, i.e., muscle mass, muscle strength and physical performance, and the overall risk of sarcopenia, among older adults. We identified 23 studies that met review inclusion criteria. The studies were diverse in terms of the design, setting, measures of diet quality, and outcome measurements. A small body of evidence suggested a relationship between “healthier” diets and better muscle mass outcomes. There was limited and inconsistent evidence for a link between “healthier” diets and lower risk of declines in muscle strength. There was strong and consistent observational evidence for a link between “healthier” diets and lower risk of declines in physical performance. There was a small body of cross-sectional evidence showing an association between “healthier” diets and lower risk of sarcopenia. This review provides observational evidence to support the benefits of diets of higher quality for physical performance among older adults. Findings for the other outcomes considered suggest some benefits, although the evidence is either limited in its extent (sarcopenia) or inconsistent/weak in its nature (muscle mass, muscle strength). Further studies are needed to assess the potential of whole-diet interventions for the prevention and management of sarcopenia.

## 1. Introduction

Sarcopenia is now widely recognised, consisting of a loss of skeletal muscle mass and physical function (muscle strength or physical performance) that occurs with advancing age [1,2,3]. It is associated with physical disability, poor quality of life and increased mortality in older adults [2] and with significant financial costs, having been estimated to increase hospitalization costs by 34% for patients aged 65 years and over [4]. Although a loss of muscle mass and decline in physical function may be expected with ageing, there is variation in the rates of decline across the population [5]. This indicates that modifiable behavioural factors such as diet could influence the development of sarcopenia. As poor diets and nutritional status are commonly reported [6,7,8,9], improving diet and nutrition may be effective for both prevention and treatment of this condition, and promoting health in later life [10].

There is significant interest in the role of dietary patterns and the effects of whole diets in predicting health, in order to take account of the collinearity between foods and nutrients and the effects of complex interactions between food constituents. The term “diet quality” is broadly used to describe how well an individual’s diet conforms to dietary recommendations and to describe how “healthy” the diet is [11,12]; often identified using principal component or factor analysis, it also includes a-priori-defined patterns, such as the Mediterranean diet. Despite using different assessment methods, there are commonalities across diet quality measures, as the “healthiness” of diets is characterised by similar foods [13]. When compared with poorer diet quality, better diet quality is characterised by higher intake of beneficial foods (e.g., fruit and vegetables, whole grains, fish, lean meat, low-fat dairy, nuts, and olive oil), but lower in energy-dense, nutrient-poor foods (e.g., refined grains, sweets and animal products that are high in saturated fats) [11,13].

Higher diet quality in older adults has been linked with various health outcomes, including to a reduced risk of common age-related diseases and to greater longevity. In general, adherence to diets of better quality, assessed by different dietary indices or a “prudent”/healthy dietary pattern, is associated with beneficial health effects; better quality diets are associated with significantly reduced risk of all-cause mortality, cardiovascular disease, cancer, type 2 diabetes, and neurodegenerative disease, as well as reduced mortality in cancer survivors [14,15,16,17]. 

Less is known about the influence of diet quality on sarcopenia (muscle mass and physical function) in older age, although there is a growing evidence base linking “healthier” diets with greater muscle strength and better physical performance outcomes in older adults [10,18]. However, much of this evidence is cross-sectional. A recent systematic review on the relationship between diet quality and successful ageing [19] concluded that with regards to physical function, there were too few longitudinal studies to draw firm conclusions, although there is growing evidence of benefits of greater adherence to a Mediterranean diet [20,21]. To the best of our knowledge no reviews have collated studies, using different definitions of diet quality, to examine effects on sarcopenia. The aim of this systematic review was to bring this evidence together and to assess the relationship between diet quality and muscle mass, muscle strength and physical performance, and sarcopenia, among older adults.

## 2. Materials and Methods

We used the methods recommended by the Centre for Reviews and Dissemination (CRD), University of York [22] and followed the Preferred Reporting Items for Systematic Reviews and Meta-Analyses (PRISMA) statement [23]. The study protocol was registered on 17 January 2017, with the PROSPERO International Prospective Register of Systematic Reviews, registration number CRD42017047597. 

### 2.1. Literature Search and Eligibility Criteria

#### 2.1.1. Inclusion and Exclusion Criteria

Eligible studies were those that reported a relationship between overall diet quality and a measure of muscle mass and/or physical function in older adults. Studies were included which met criteria in terms of the sample of people investigated, the exposure, the outcomes and the study design.

To be included in this review, studies were required to meet the following criteria: (1) be published in a peer-reviewed journal, with full-text availability in English; (2) the study participants were aged 50 years or older, or aged 50 years or older at study baseline for longitudinal studies (in order for a study to be included in this review, all participants needed to be over 50 years), and we included studies concerning specific patient groups such as overweight older adults or those with type 2 diabetes; (3) the study reported an analysis of the relationship between diet quality as measured using dietary patterns (including a priori dietary indices or *a posteriori* (or data-driven) methods [11]), or a measure of dietary variety, and an appropriate outcome measure as specified below; or an intervention study that reported the effect of following recommendations for a “healthy” diet (leading to improvements in the overall quality of diet) on an appropriate outcome measure; (4) the study included at least one physical function outcome measure of the following: muscle mass, muscle strength, physical performance, or sarcopenia (see Table 1 for further details); (5) observational studies (cohort, case-control, cross-sectional), as well as randomised controlled trials with relevant data. The exclusion criteria included: (1) the study included age groups younger than 50 years; (2) the study combined diet quality with other lifestyle measures into a “lifestyle score” (except where associations with diet quality was reported separately); (3) the study evaluated intake of individual nutrients or single foods or food groups only; (4) the study only included a subjective measure of the physical function outcome, with no objective measure available; (5) the study outcome was protein synthesis, muscle fibre hypertrophy or biochemical properties of muscle.

#### 2.1.2. Search Strategy

An information specialist provided assistance in generating relevant search terms and performing the literature search. Eight databases, namely MEDLINE, Embase, Web of Science Core Collection, CINAHL, AMED, Cochrane Database of Systematic Reviews, Cochrane Central Register of Controlled Trials, and DARE via Cochrane Library, were searched for relevant articles using both MeSH (Medical Subject Headings) terms and free-text terms related to diet quality and muscle outcomes. A sample of the search strategy and search terms that were used for this research (applied in the MEDLINE database) are detailed in a supplementary table (see Table S1 in the Supplementary Materials published online). The search terms (including terms relating to ageing) were combined using Boolean operators (“AND”, “OR”), and filters were used to limit the results to those in the English language and in humans. The search was performed in August 2016 and no date restrictions were applied. We considered studies conducted in any part of the world and the setting was noted during the data extraction process. 

### 2.2. Study Selection

**Figure 1:**
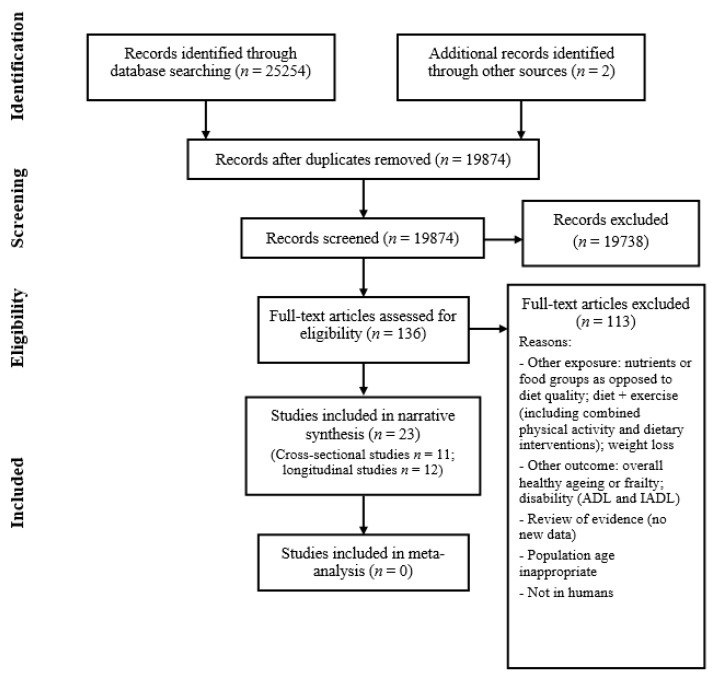
Flow diagram summary of articles identified in search and showing the selection of studies for inclusion in the review.

Figure 1 shows how studies were identified and selected for inclusion in the review. The database search yielded 25,254 results and discussions with experts, followed by hand searching, yielding another two studies. A total of 5382 duplicate articles were removed, leaving a total of 19,874 articles to screen titles and abstracts. Two authors (I.B. and C.S.) independently screened these records with 19,738 articles being excluded at this stage. Full-text articles were retrieved for 136 records and both authors assessed these for eligibility. This led to the identification of 23 papers which were eligible for inclusion in the review. In cases of disagreement about a study’s suitability, a third author (J.B.) was consulted. I.B. and C.S. also screened the reference lists of the included papers for any further potentially relevant articles, although none were found.

### 2.3. Data Extraction and Assessment of Risk of Bias

The two reviewers, I.B. and C.S., working independently, extracted relevant data from each included article and assessed risk of bias using established criteria for observational studies, following the methods recommended by the CRD and adapted from a standard assessment tool [24]. The form was piloted on the included studies. The reviewers assessed the risk of bias of each study in relation to the review question using a set of 10 criteria, and recorded the results electronically (see Table S2 in the Supplementary Materials published online for details on the criteria and scoring system used to assess risk of bias of studies included in this review). These addressed areas including study setting, design and population, whether the exposure and outcome measurements were reliably obtained, losses to follow-up and the appropriateness of analyses presented. Regarding the confounding factors adjusted for in the analyses, we decided a priori which were important and assessed studies on the basis of how many of these were adjusted for in the analyses (the following factors were considered as important confounding factors: age, gender, physical activity, ethnicity, socioeconomic status/education, co-morbidities, smoking status). I.B. and C.S. independently carried out the quality assessment of each paper, and any discrepancies in scoring were resolved by mutual discussion or through discussion with J.B. An overall risk of bias rating was assigned to each study based on the quality score; studies were classified as either “high risk” (total score −9 to −3); “medium risk” (−2 to +3); or “low risk” of bias (+4 to +10) (see Table S2 in the Supplementary Materials for further details).

### 2.4. Data Synthesis

We carried out a narrative synthesis of study findings and considered the scope for meta-analysis. 

## 3. Results

Twenty-three studies met review inclusion criteria. Studies are grouped according to outcome in Table 2 and their characteristics are shown in Table 3, also presented by outcome, first describing longitudinal and then cross-sectional studies. There were 11 cross-sectional and 12 longitudinal studies. Sample sizes ranged from 98 to 5350 participants. Almost half of the studies (*n* = 11) had over 1000 participants. Most studies (*n* = 21) were set in the community, with one set in a nursing home and another including participants from either the community or care facilities. Most of the studies (*n* = 16) had participants whose mean age ranged between 65 and 75 years, with five studies featuring mean ages > 75 years and only two studies having participants whose mean age was below 65 years.

Diet quality as an exposure was measured using different methods. Eight studies used *a posteriori* or data-driven methods, namely principal component analysis or factor analysis [25,26,27,28,29,30], and cluster analysis [31,32], to assess diet quality. Seventeen of the included studies included a priori measures of diet quality (i.e., diet indices) [26,29,33,34,35,36,37,38,39,40,41,42,43,44,45,46,47]; 15 different diet indices were used (Table 3) (dietary variety score, *n* = 1; fruit and vegetable variety score, *n* = 1; Mediterranean-type diet score (mMedTypeDiet), *n* = 1; Canadian Healthy Eating Index (C-HEI), *n* = 1; Dietary Variety Score (DVS), *n* = 2; Mediterranean diet score (MDS), *n* = 6; Alternative Healthy Eating Index (AHEI), *n* = 1; Nordic diet score (NDS), *n* = 1; Mediterranean Diet Adherence Screener (MEDAS), *n* = 1; Healthy Eating Index-2005 (HEI-2005), *n* = 1; alternate MED score, *n* = 1; Mediterranean Style Dietary Pattern Score (MSDPS), *n* = 1; Healthy Eating Index (HEI), *n* = 1; Healthy Diet Indicator (HDI), *n* = 1; Diet Quality Index-International (DQI-I), *n* = 1), with four studies including multiple indices [29,33,42,47] and two studies including both indices and *a posteriori* methods in their analyses [26,29]. The most common a priori method used was assessment of adherence to a Mediterranean diet.

The outcomes considered, namely muscle mass, muscle strength, physical performance, and sarcopenia, were assessed using various objective tests or measurements (the outcomes considered were decided by the inclusion criteria—see Table 1). Muscle mass outcomes included appendicular lean mass (ALM) or appendicular skeletal muscle mass (ASM) [46]; ALM/Wt (Weight) [31]; ALM/BMI (body mass index) [34]; ALM/FM (fat mass) [34]; percentage lean mass [47]; mean arm muscle area [33]; and thigh muscle area [33]. Muscle strength outcomes included handgrip strength (most commonly assessed) [25,28,32,35,41,42,44,45,46]; knee extensor strength [35,36,43]; and elbow flexor strength [35]. Physical performance outcomes comprised walking speed (most commonly assessed) [26,28,38,40,41,42,43,44,46]; Short Physical Performance Battery (SPPB) [37,45,47]; Timed Up-and-Go (TUG) test [32]; chair–rise test (sit–stand chair rises) [27]; balance test [27]; and the Senior Fitness Test (SFT) battery [39]. Sarcopenia was defined in one study according to the Asian Working Group for Sarcopenia (AWGS) algorithm [29], and in the other according to the European Working Group on Sarcopenia in Older People (EWGSOP) criteria [30]. More than half of the studies (*n* = 13) used cut-off values for muscle mass, strength or function, and ten of the included studies used continuous scales to describe muscle outcomes.

Eight of the included studies were classified as having a low risk of bias in relation to our research question, while only two were deemed to have a high risk of bias (Table 2). Over half of the studies (*n* = 13) were classified as having a medium risk of bias (see Table S3 in the Supplementary Materials published online for a summary table of risk of bias for all studies included in the review).

The synthesis of study findings is presented by outcome in the following order: muscle mass, strength, physical performance, and sarcopenia.

### 3.1. Muscle Mass

Of the five studies that included muscle mass as an outcome (Table 2), four showed a positive association with diet quality. Four were cross-sectional [31,33,34,47]; none had a low risk of bias, and two had a high risk of bias [33,47]. Of the five studies, one used *a posteriori* methods to assess diet quality [31], and four used dietary indices or a priori measures of diet quality [33,34,46,47]. One of the studies was European [34], one was from the US [33], one was Australian [47], and two were from Asia [31,46].

A cross-sectional study [33] found that in women, a higher fruit and vegetable variety score was associated with higher mid-arm muscle area. Another cross-sectional study from Korea [31] found that a westernized dietary pattern was associated with a markedly increased abnormality of muscle mass (ASM/Wt (kg)) (authors defined abnormality of ASM/Wt as being less than the value of a young reference group, aged 20–39 years), compared to a more traditional pattern. However, no association was observed for a dietary pattern characterised by a higher consumption of meat and alcohol, and muscle mass. Another cross-sectional study [34] found that better adherence to a Mediterranean diet was associated with better muscle mass outcomes in women, but not in men. An Australian cross-sectional study [47] did not find an association between lean mass and the HEI (Healthy Eating Index) score; however, in women, there was a weak association between a higher HDI (Healthy Diet Indicator) score and higher percentage of lean mass, which disappeared when adjusted for age. The single longitudinal study [46] found that better diet quality (greater dietary variety) was not significantly associated with changes in either lean body mass or appendicular lean mass. 

To summarise, there is a small body of mainly cross-sectional evidence regarding the relationship between diet quality and muscle mass, suggesting a possible relationship between healthier diets and better muscle mass outcomes in older people, especially in women. Overall, however, the evidence for an association between diet quality and muscle mass is weak, especially given the relatively lower quality of studies in this group.

### 3.2. Muscle Strength

Eleven of the included studies examined muscle strength as an outcome (Table 2), of which five showed a positive association with diet quality. Four were cross-sectional studies [25,43,44,45] and five had a low risk of bias [25,28,42,43,44]. Of the 11 studies, three used *a posteriori* methods to assess diet quality [25,28,32], and eight used a priori measures of diet quality [35,36,41,42,43,44,45,46]. Seven of the studies were European [25,28,32,41,42,44,45], two were North American [35,43], and two were from Japan [36,46].

One of the cross-sectional studies [25] found a healthier pattern of eating to be independently associated with higher handgrip strength in women but not in men. Another cross-sectional study [43] found that higher total HEI-2005 scores were associated with greater knee extension strength, however this association was no longer statistically significant after adjustment for physical activity. Two cross-sectional studies [44,45] found no statistically significant associations between higher adherence to a Mediterranean dietary pattern and handgrip strength. In a longitudinal study [32], dietary patterns high in red meats, potato and gravy, or butter were associated with lower grip strength and greater decline in grip strength in men, however, no association was observed in women. A longitudinal Japanese study [46] found greater dietary variety to be associated with lower risk for future declines in grip strength. Conversely, three other longitudinal studies [28,41,42] found no statistically significant associations between diet quality and handgrip strength. Another longitudinal study [35] showed no significant association between diet quality measured using the C-HEI (Canadian Healthy Eating Index) at baseline and maintenance of three measures of muscle strength. Similarly, a longitudinal study of Japanese women [36], found no significant relationship between diet quality (dietary variety) and knee extension strength.

To summarise, few studies have found positive associations between diet quality and muscle strength, and there is limited evidence for a link between “healthier” diets and lower risk of declines in muscle strength. There was a suggestion that the effects of diet on muscle strength might be different for men and women, however the evidence was inconsistent. The quality of studies was fairly good, given that almost half the studies had a low risk of bias and the others had a medium risk of bias. Overall, however, the current evidence regarding the relationship between diet quality and muscle strength is inconsistent.

### 3.3. Physical Performance

Of the 15 studies that looked at physical performance (Table 2), 14 showed a positive association with diet quality. Six studies were cross-sectional [27,40,43,44,45,47]; and six had a low risk of bias [28,38,39,42,43,44]. Of the 15 studies, four used *a posteriori* methods to assess diet quality [26,27,28,32], and 12 used a priori measures [26,37,38,39,40,41,42,43,44,45,46,47]. Ten of the studies were European [26,27,28,32,37,39,41,42,44,45], three of the studies were from the US [38,40,43], one was Australian [47], and one from Japan [46].

Two cross-sectional studies [40,44] found an association between higher adherence to a Mediterranean dietary pattern and faster walking speed (better physical performance). Another cross-sectional study [45] found that high adherence to a Mediterranean dietary pattern was associated with a higher SPPB score (better physical performance). A cross-sectional study [43] found that older adults with higher HEI-2005 scores had a faster walking speed, although this association was no longer statistically significant after adjustment for physical activity. On the other hand, another cross-sectional study [47] found no significant association between HEI score and SPPB score in either men or women (separately), but in men there was a weak association between a higher HDI score and better SPPB. Another study [27] did not find any independent associations between a “prudent” dietary pattern and physical performance measures. A longitudinal study [28] found that greater adherence to a “Westernized” diet pattern was associated with increased risk of slow walking speed, after a follow-up period of three and a half years. A greater adherence to a “prudent” diet pattern showed a statistically non-significant association with a lower risk of slow walking speed. Similarly, another longitudinal study [26] found that participants with greater adherence to the “Western-type” dietary pattern were more likely to have lower walking speed; the study did not find an association for the “healthy-foods” dietary pattern, or for adherence to the AHEI (Alternative Healthy Eating Index). A longitudinal study [42] found that a higher Mediterranean diet score was associated with reduced risk of slow walking after the follow-up period. Another longitudinal study [46] found that greater dietary variety was associated with lower risk for future declines in walking speed. Three other longitudinal studies [37,38,41] reported consistent associations between higher adherence to a Mediterranean diet at baseline and better physical performance (smaller decline) after follow-up periods ranging from three to nine years (in two studies, measured as walking speed, and in one using the SPPB), even when adjusting for physical activity in two of them [37,38]. In a longitudinal study [32], men with dietary patterns high in red meats and women with dietary patterns high in butter had worse physical performance (slower Timed Up-and-Go Test) than those with a “low meat” pattern, but similar rates of decline. Another longitudinal study [39] found that for women a healthy Nordic diet predicted better physical performance (SFT) at 10-year follow-up; however, no such association was observed in men. 

To summarise, there is a sizeable body of longitudinal evidence regarding the relationship between diet quality and physical performance, which shows consistent evidence for a link between “healthier” diets and smaller declines in physical performance. The evidence suggests that there may be differences in the effects of diet on performance for men and women, although these gender differences were inconsistent across the studies reviewed. The quality of studies was fairly good, given that the majority of studies had a medium risk of bias and six had a low risk of bias. Overall, the current observational evidence of a positive relationship between diet quality and physical performance is strong.

### 3.4. Sarcopenia

Both of the studies that looked at sarcopenia (Table 2) showed an association with diet quality; one had a low risk of bias [29] and the other a medium risk of bias [30]. Both of the studies used data-driven methods to measure diet quality and one also used dietary indices [29]. A cross-sectional Iranian study [30] found that individuals with greater adherence to a Mediterranean diet pattern had a lower odds ratio for sarcopenia. A longitudinal study from China [29] found that a higher “vegetables–fruits” dietary pattern score was associated with lower likelihood of prevalent sarcopenia in older men; however, no such associations were observed in women. Although the study [29] found no association between adherence to a Mediterranean diet and odds of sarcopenia, it found that a higher DQI-I (Diet Quality Index—International) score was associated with lower likelihood of prevalent sarcopenia in older men, although no such association was observed in women. Furthermore, no significant associations were found between any of the diet quality measures and four-year incident sarcopenia in either gender.

To summarise, the small body of evidence for the relationship between diet quality and sarcopenia points to a possible association between healthier diets and lower likelihood of sarcopenia in older people. There is, however, a lack of longitudinal evidence for a relationship. The quality of studies was fairly good, given that one had a low risk of bias and the other a medium risk. Overall, there is some cross-sectional evidence for a link between “healthier” diets and lower odds of sarcopenia.

We could not carry out a meta-analysis for any of the groups of studies because the definitions of both the exposure (measures of diet quality) and outcomes (measures of muscle mass, muscle strength, physical performance and sarcopenia) varied widely between studies.

## 4. Discussion

We systematically assessed the evidence regarding the relationship between diet quality and muscle mass, muscle strength and physical performance, and sarcopenia in later life. To the best of the authors’ knowledge this is the first study to systematically review this body of evidence. We found 23 studies of older adults (≥50 years) in which the association of overall diet quality and relevant outcomes was examined. The studies were diverse in terms of the design (cross-sectional vs. longitudinal, and the duration of follow-up), setting, participants included (ages varied from early old age to the very old), measures of diet quality, outcome measurements, as well as confounding factors adjusted for in the statistical models. These discrepancies could potentially contribute to some of the heterogeneity in the results. 

Overall, there is a small body of mainly cross-sectional evidence suggesting a possible relationship between healthier diets and better muscle mass outcomes in older people, although, on the whole, the current evidence is fairly weak. There is limited evidence for a link between “healthier” diets and lower risk of declines in muscle strength, and overall the evidence is inconsistent. In contrast, there is a sizeable body of longitudinal evidence providing consistent evidence of a link between “healthier” diets and smaller declines in physical performance; overall, the current observational evidence for a positive relationship between diet quality and physical performance is strong. Overall, there is a small body of cross-sectional evidence pointing to a possible association between healthier diets and lower likelihood of sarcopenia in older people. There is, however, a lack of longitudinal evidence for a relationship. Some of the evidence suggests that there may be differences between men and women in terms of the effects of diet quality on both muscle strength and physical performance, although these findings were inconsistent and the overall message on differences by gender was not clear.

A recently published longitudinal study by Perälä and colleagues [52], not included in this review, provides further evidence of the benefits of diet quality for muscle strength (both grip strength and knee extensor strength). The authors found that in women, adherence to a healthy Nordic diet was associated with greater muscle strength measured 10 years later.

In general, “healthier” diets are characterised by greater fruit and vegetable consumption, greater consumption of wholemeal cereals and oily fish, which indicate higher intakes of a range of nutrients and dietary constituents that could be important for health, including for muscle function, such as higher intakes of vitamin D and n-3 long-chain polyunsaturated fatty acids (LCPUFAs), higher antioxidant and protein intakes [18]. There is evidence for a link between differences in nutrient intake and status and the components of sarcopenia, with the most consistent associations found for protein, vitamin D, antioxidant nutrients and long-chain polyunsaturated fatty acids [18]. Protein intake has been recognised as one of the main anabolic stimuli for muscle protein synthesis [18]. There is growing evidence for benefits of supplementation with vitamin D to preserve muscle mass, strength and physical function in older age and to prevent and treat sarcopenia, and it could be that supplementation with vitamin D in combination with other nutrients might be important [18]. Sarcopenia is considered to be an inflammatory state driven by cytokines and oxidative stress; an accumulation of reactive oxygen species may lead to oxidative damage and likely contribute to losses of muscle mass and strength [10]. Omega-3 LCPUFAs have potent anti-inflammatory properties, and variations in intake could be of importance [10]. Aside from effects on inflammation, these fatty acids could also have direct effects on muscle protein synthesis [18]. “Healthier diets” are also higher in plant phytochemicals, such as polyphenols, which could have antioxidant and anti-inflammatory effects on muscle mass and function [18].

Although we did not find evidence for a longitudinal relationship between diet quality and sarcopenia in this review, there is evidence from a recent systematic review that better diet quality is associated with lower risk of prevalent, as well as future, frailty [53]. Nevertheless, of the 19 studies that were included in that review, only three studies examined the relationship between overall diet quality and frailty. The identification of frailty was based on the frailty phenotype described by Fried et al. [54], in two of the three studies, and on the FRAIL scale [55] in the other study. Some of the Fried frailty assessment components, namely muscle strength (grip strength) and physical performance (walking time), are common to sarcopenia. However, the FRAIL frailty scale is based on self-reported criteria (including difficulty walking), so the relationship with sarcopenia is less clear. A recent systematic review on diet quality and successful ageing [19] did not include any studies that investigated the link between diet quality measured using data-driven methods and physical function. The review did include studies that assessed the relationship between dietary indices and physical function in older adults (using both report-based and objective measures). Although there was a lack of longitudinal studies, the findings suggested a relationship between healthier diets and better physical function. Two recent systematic reviews investigated the relationship between adherence to a Mediterranean diet and musculoskeletal–functional outcomes; one focused on musculoskeletal health (including bone and muscle outcomes, specifically sarcopenia incidence or combined outcomes) in all ages [20], while the other investigated the association between a Mediterranean diet and frailty, functional disability and sarcopenia in older people [21]. The review findings indicate growing evidence of benefits of greater adherence to a Mediterranean diet, although they conclude that further research is needed to understand the relationship between a Mediterranean diet and sarcopenia and musculoskeletal health.

The studies included in the present review were all observational and most of them were from high-income countries (the majority of the studies were from countries in Europe or North America, four were from Asia, and one was from Australia). These are limitations of the current evidence-base and there is a need for more intervention studies, especially from lower-income populations, to improve our understanding of effects of diet quality on physical function. In most of the studies, diet was only assessed at one time point (baseline) therefore, any changes in diet during the follow-up period were not captured. Although different methods were used across the studies to assess dietary intake, different dietary assessment methods have been shown to define dietary patterns in a comparable manner [56,57,58]. The diversity in methods of diet quality assessment, e.g., different statistical techniques and numerous dietary indices with differing scoring approaches, should be noted; however, the core tenets of these measures are similar since the “healthiness” of diets is generally characterized by similar foods [11,13]. Around half of the included studies (*n* = 12) adjusted for energy intake in their analyses, and a limitation of this review is that energy intake was not considered as one of the important confounders when quality assessing the studies. A further limitation is that diverse measures of effect sizes were used across the studies, making it difficult to draw any firm conclusions about effect size (the smallest effect size for the relationship between diet quality and physical performance was a regression coefficient of 0.06 and the largest was an odds ratio of 1.85). This systematic review employed a comprehensive search strategy, in which eight databases were systematically searched, and supplemented by hand searching and contact with experts. The approach to study selection, data extraction and quality assessment followed guidance for best practice in systematic reviews, and findings are reported according to the PRISMA guidance. Another strength is that this review provides evidence for the benefits of a range of diets on musculoskeletal–functional outcomes in older people, adding to the existing evidence base that links overall diet quality with health outcomes in later life, including all-cause mortality [16,59] and chronic disease [60]. A common limitation of systematic reviews is publication bias. Although we have attempted to mitigate against this by contacting experts and hand searching, we did not identify any unpublished analyses and it remains a potential limitation of this work.

## 5. Conclusions

This systematic review provides observational evidence to support the importance of diets of adequate quality to protect physical performance in older age. Findings for muscle mass, muscle strength and sarcopenia are also suggestive of a link with “healthier” diets, although there are gaps in the evidence base and further studies are needed. The balance of the existing observational evidence suggests that the potential of intervention studies that take a whole-diet approach, leading to changes in intakes of a range of foods and nutrients, should be explored as strategies for the prevention and/or management of age-related losses in muscle mass and physical function. Further intervention studies are needed, especially from lower-income countries and populations, to improve our understanding of effects of diet quality on sarcopenia and its components.

## Figures and Tables

**Table 1:** Types of measures considered for relevant outcomes, namely muscle mass, strength, physical performance, and sarcopenia.

Outcome	Muscle Mass	Muscle Strength	Physical Performance	Sarcopenia
Acceptable measures	Anthropometry ^a^ Dual energy X-ray absorptiometry (DXA) Bio impedance analysis (BIA) Computed tomography (CT) Magnetic resonance imaging (MRI)	Handgrip strength Quadriceps strength Muscle quality index	Short Physical Performance Battery (SPPB) Gait/walking speed Timed get-up-and-go test Balance Stair climb power test	Combined outcomes of muscle mass, muscle strength or physical performance

^a^ Includes mid-arm circumference and triceps skinfold measures to determine mean arm muscle area (MAMA).

**Table 2:** Summary of systematic review studies by outcome.

Muscle Mass	Muscle Strength	Physical Performance	Physical Performance + Muscle Strength	Physical Performance + Muscle Strength + Muscle Mass	Physical Performance + Muscle Mass	Sarcopenia
Bernstein, 2002 [33]	Rahi, 2014 [35]	Milaneschi, 2011 [37]	Talegawkar, 2012 [41]	Yokoyama, 2017 [46]	Smee, 2015 [47]	Chan, 2016 [29]
Cross-sectional	Longitudinal; FU: 3 years	Longitudinal; FU: 3 years, 6 years and 9 years	Longitudinal; FU: 3 years and 6 years	Longitudinal; FU: 4 years	Cross-sectional	Longitudinal; FU: 4 years
Risk of bias: High	Risk of bias: Medium	Risk of bias: Medium	Risk of bias: Medium	Risk of bias: Medium	Risk of bias: High	Risk of bias: Low
Oh, 2014 [31]	Kojima, 2015 [36]	Shahar, 2012 [38]	León-Muñoz, 2014 [42]			Hashemi, 2015 [30]
Cross-sectional	Longitudinal; FU: 4 years	Longitudinal; FU: 8 years	Longitudinal; FU: 3.5 years			Cross-sectional
Risk of bias: Medium	Risk of bias: Medium	Risk of bias: Low	Risk of bias: Low			Risk of bias: Medium
Nikolov, 2016 [34]	Robinson, 2008 [25]	Akbaraly, 2013 [26]	León-Muñoz, 2015 [28]			
Cross-sectional	Cross-sectional	Longitudinal; FU: 16 years	Longitudinal; FU: 3.5 years			
Risk of bias: Medium	Risk of bias: Low	Risk of bias: Medium	Risk of bias: Low			
		Perälä, 2016 [39]	Granic, 2016 [32]			
		Longitudinal; FU: 10 years	Longitudinal; FU: 5 years			
		Risk of bias: Low	Risk of bias: Medium			
		Martin, 2011 [27]	Xu, 2012 [43]			
		Cross-sectional	Cross-sectional			
		Risk of bias: Medium	Risk of bias: Low			
		Zbeida, 2014 [40]	Bollwein, 2013 [44]			
		Cross-sectional	Cross-sectional			
		Risk of bias: Medium	Risk of bias: Low			
			Fougère, 2016 [45]			
			Cross-sectional			
			Risk of bias: Medium			

FU: length of follow-up, for longitudinal studies.

**Table 3:** Characteristics of studies included in the systematic review.

First Author, Year	Setting	Study Participants	Study Design	Measure of Physical Function	Assessment of Dietary Intake + Measure of Diet Quality (DQ)	Association of Outcome with Exposure	Risk of Bias *^a^*
**Muscle Mass**							
Bernstein, 2002 [33]	The Boston FICSIT Study, Boston, MA, USA	98 men and women older than 70 years (aged 72 to 98 years) were recruited among residents of a nursing home Mean ± SD age: 87.1 ± 0.6 years	CS	Mean arm muscle area (MAMA) was calculated and thigh muscle area was measured using computerized tomography (CT) scanning.	3-day weighed food records on 3 consecutive days of the week Dietary indices: 2 dietary variety scores.		−4: High
					(1) Dietary variety score equal to the number of different foods eaten over 3 days.	MAMA approached a significant relationship with dietary variety score (*p* = 0.06) in both men and women. No association with thigh muscle area was reported.	
					(2) Fruit and vegetable variety score equal to the number of different fruits and vegetables consumed over the 3 days.	In women, high fruit and vegetable variety score only was associated with higher MAMA (β = 2.94) (*p* ≤ 0.03). Thigh muscle area was tested and no significant association was found. Conclusions: Results suggest that a highly varied diet in elderly nursing home residents is associated with higher MAMA.	
Oh, 2014 [31]	The KNHANES 2011, Korea	1435 non-institutionalized Korean people who were aged 65 years or more Mean ± SD age: not given	CS	Appendicular skeletal muscle mass (ASM) was measured by DXA. ASM was defined as the sum of lean soft tissue masses for the arms and legs, after the method of Heymsfield et al. [48].	Single 24-h dietary recall Data-driven: cluster analysis.		+3: Med
					(1) “Traditional Korean” dietary pattern ^b^, in which consumption of white rice accounted for 76% of total energy intake.	Compared with the “Traditional Korean” pattern, the “Westernized Korean” pattern was associated with a 74% increased abnormality of ASM/Wt (kg) by logistics analysis.	
					(2) “Meat and Alcohol” dietary pattern, with a higher consumption of meat and alcohol.	No association was observed.	
					(3) “Westernized Korean” dietary pattern, based on a rice and vegetable diet but characterized by a variety of food groups such as other grains, fruit, bread, eggs, fish, milk, and alcohol.	See above. Conclusions: A “Westernized Korean” pattern was associated with a markedly increased abnormality of muscle mass, compared to the “Traditional Korean” pattern.	
Nikolov, 2016 [34]	BASE-II, Berlin, Germany	1509 community-dwelling, well-functioning older men and women between 60 and 80 years. Mean ± SD age: 68.2 ± 3.7 years	CS	Body composition was assessed by using DXA. ALM was calculated as the sum of bone-free lean mass of arms and legs and related to height and weight (ALM/BMI). The proportion of ALM to whole body fat mass (FM) was defined as the ALM/FM ratio.	Self-administered EPIC-FFQ. Dietary indices: Adherence to a Mediterranean dietary pattern was assessed using the Mediterranean-type diet score (mMedTypeDiet) suggested by Grosso and colleagues.	A higher adherence to the mMedTypeDiet was associated with higher ALM/BMI in women and better ALM/FM ratio when compared to a medium and a low diet quality. No significant association was found in men. Conclusions: Higher adherence to a Mediterranean-style diet was associated with a positive effect on ALM/BMI in women.	+3: Med
**Muscle Strength**							
Rahi, 2014 [35]	Secondary analysis of the NuAge cohort, QC, Canada.	156 community-dwelling men and women with type 2 diabetes Mean ± SD age: at baseline 74.6 ± 4.2 years	LS	Handgrip, knee extensor and elbow flexor strengths, were measured at recruitment and at the 3-year follow-up. Crude change was calculated by subtracting values at recruitment from values at the 3-year follow-up. In order to show the yearly MS decline, the percentage relative change per year was adjusted for baseline value.	Three non-consecutive 24-h dietary recalls (on two randomly-chosen weekdays and one weekend day). Dietary indices: DQ was evaluated at recruitment using the Canadian Healthy Eating Index (C-HEI).	There was no effect of DQ at baseline on maintenance of the three measures of muscle strength, in either males or females. Likewise, DQ, which was dichotomized based on the median or categorized into quartiles, showed no significant effects on MS maintenance. Conclusions: DQ alone had no effect on MS maintenance in this sample of diabetic older men and women. However, when good DQ was combined with stable or increased PA, MS losses were minimal in diabetic older males over the 3-year follow-up, despite some discordance between changes in MS in the upper and lower extremities.	+2: Med
Kojima, 2015 [36]	Itabashi Ward of Tokyo, Japan	575 community-dwelling women from the Itabashi Ward of Tokyo Mean ± SD age: ages ranged between 75 and 85 years (78.07 ± 2.56) in 2008 and between 78 and 89 years (82.07 ± 2.55) in 2012	LS	Isometric knee extension strength (KES, in N) was measured in the dominant leg using a hand-held dynamometer incorporated into a custom-made frame.	Participants were asked closed-ended questions about intake frequencies of 10 food groups Dietary indices: A DVS, an index of dietary variety introduced by Kumagai et al. [49], was calculated.	There was no significant cross-sectional relationship between KES and DVS. Longitudinal analysis showed that except for 3 food groups, no lifestyle-related variables at baseline were related to changes in KES over 4 years. Conclusions: The age-related decline in muscle strength was lower in people who frequently ate soy products or green and yellow vegetables, but no association was found with DVS.	+1: Med
Robinson, 2008 [25] *	HCS, UK	2983 community-dwelling men and women aged 59 to 73 years Mean ± SD age: Men: 65.7 ± 2.9 years; Women: 66.6 ± 2.7 years	CS	Maximum grip strength was measured using a handgrip Jamar dynamometer.	Administered FFQ based on EPIC questionnaire, pertaining to 3-month period preceding the interview. Data-driven: PCA. Using PCA, a “prudent” dietary pattern ^c^ was identified.	Men and women with high prudent diet scores had higher grip strength. In men, the association was no longer evident when fatty fish consumption was accounted for. In women, independent associations between grip strength and prudent diet score and fatty fish consumption remained, although the size of the effect was markedly reduced (regression coefficient of 0.17, 95% CI = 0.00 to 0.34 kg per unit change in score, *p* = 0.044). Conclusions: Whilst a healthier pattern of eating was associated with higher grip strength, this effect was at least partly explained by more prudent diets also being characterised by greater consumption of fatty fish.	+5: Low
**Physical Performance**							
Milaneschi, 2011 [37] *	InCHIANTI (Invecchiare in Chianti), study, Tuscany, Italy	Older men and women: 705 participants had available data on lower body mobility at 3-year follow-up, 614 at 6-year follow-up and 486 at 9-year follow-up. Mean ± SD age: at baseline 74.1 ± 6.8 years	LS	Lower extremity function was measured at baseline, and at the 3-, 6- and 9-year follow-up visits using the SPPB, which was derived from three objective tests: 4-m walking speed, repeated chair rises and standing balance in progressively more challenging positions.	FFQ created for EPIC, validated in this population. Dietary indices: Adherence to a Mediterranean dietary pattern was assessed using the MDS by Trichopoulou et al. [50].	At baseline, higher adherence to Mediterranean diet was associated with better lower body performance. Participants with higher adherence experienced less decline in SPPB score, which was of 0.9 points higher (*p* < 0.0001) at the 3-year-follow, 1.1 points higher (*p* = 0.0004) at the 6-year follow-up and 0.9 points higher (*p* = 0.04) at the 9-year follow-up compared to those with lower adherence. Among participants free of mobility disability at baseline, those with higher adherence had a lower risk (HR (hazard ratio) = 0.71, 95% CI = 0.51–0.98, *p* = 0.04) of developing mobility disability (defined as SPPB ≤ 9 points). Conclusions: High adherence to a Mediterranean-style diet is associated with a slower decline of mobility over time in community-dwelling older persons.	+2: Med
Shahar, 2012 [38]	Health, Aging, and Body Composition cohort study, USA	1201 participants Mean ± SD age: at baseline 74.6 ± 2.9 years (middle category of MD score)	LS	Performance-based evaluations included usual and rapid walking speed assessed over a 20-metre course.	Administered FFQ Dietary indices: Adherence to a Mediterranean dietary pattern was assessed using the MDS by Trichopoulou et al. [50].	Higher MD adherence was an independent predictor of less decline in usual 20 m walking speed (*p* = 0.049). The effect decreased after adding total body-fat-percent to the model (*p* = 0.134). Similar results were observed for MD .adherence and rapid 20 m walking speed; the association remained significant after adjustment for total body-fat-percent (*p* = 0.012). Conclusions: Walking speed over 8 years was faster among those with higher MD adherence at baseline. The differences remained significant over 8 year, suggesting a long-term effect of diet on mobility performance with aging.	+4: Low
Akbaraly, 2013 [26]	Whitehall II study (London-based office staff), UK	5350 men and women aged 60 years or older at the final follow-up Mean ± SD age: at baseline 51.3 ± 5.3 years	LS	Walking speed over a 8-feet walking course.	Semi-quantitative FFQ Data-driven: PCA. Two dietary patterns were identified.		+2: Med
					(1) “Healthy-foods” dietary pattern.	No association was reported	
					(2) “Western-type” dietary pattern.	Participants in the highest tertile of “Western-type” dietary pattern, compared with those in the bottom tertile, were more likely to have poorer musculoskeletal functioning (OR (odds ratio) = 1.45; 95% CI = 1.14–1.84).	
					Dietary indices: Adherence to the Alternative Healthy Eating Index (AHEI) was calculated.	No association was reported Conclusions: Avoidance of “Western type foods” might increase the possibility of achieving older ages with better musculoskeletal functioning (faster walking speed).	
Perälä, 2016 [39]	Helsinki Birth Cohort Study, Finland	1072 men and women Mean ± SD age: 61.3 ± 0.2 (SE) years	LS	Physical performance was assessed using the validated Senior Fitness Test (SFT) battery *^d^*.	Self-administered FFQ pertaining to the previous 12 months Dietary indices: An a priori-defined Nordic diet score (NDS) was calculated, as a measure of a healthy Nordic diet.	In a fully adjusted model, the overall Senior Fitness Test (SFT) score was 0.55 (95% CI = 0.22, 0.88) points higher per 1 unit increase in the NDS. Women in the highest fourth of the NDS had on average 5 points higher SFT score compared with those in the lowest fourth (*p* for trend 0.005). No such association was observed in men. Women with the highest score had 17% better result in the walk test, 16% better arm curl and 20% better chair stand results compared with those with the lowest score (all *p* values < 0.01). Conclusions: The study indicates that among women a healthy Nordic diet predicts better physical performance, and especially better aerobic endurance and upper- and lower-body strength 10 years later.	+5: Low
Martin, 2011 [27] *	HCS, UK	628 community-dwelling men and women Mean ± SD age: 68.0 ± 2.5 years	CS	Participants completed a short physical performance battery. This included measures of time taken to complete a 3-m customary pace walk and 5 sit–stand chair rises; balance performance was assessed by measurement of one-legged timed standing balance.	Administered FFQ pertaining to 3-month period preceding the interview Data-driven: PCA. A “prudent” dietary pattern was identified ^c^.	In men, no independent associations were found between 3-m walk time and diet. For women, a higher prudent diet score was associated with shorter 3-m walk time (*p* = 0.016), although this association was not robust to adjustment for confounding factors. In men, there were no associations between diet and chair-rise time. Among the women, univariate comparisons showed that shorter chair-rise times were associated with higher prudent diet scores (*p* = 0.011). However, this association was not robust to adjustment. Higher prudent diet scores in the women were associated with better balance (*p* = 0.033) but this was not robust to adjustment for confounders. This association was not observed in men. Conclusions: There were no independent associations between the dietary pattern and physical performance.	+3: Med
Zbeida, 2014 [40] *	US NHANES and the Israeli MABAT ZAHAV survey ^e^	NHANES: 2791 people aged 60 years and older. Mean age: 71.2 years.	CS	Observed timed 20-feet walk.	24-h multiple-pass dietary recall interview on a random day of the week. Dietary indices: Adherence to a Mediterranean dietary pattern was assessed using the MDS, similar to that constructed by Trichopoulou et al. [50].	MDS (high vs. low) was associated with faster walking speed after adjusting for confounders in a logistic regression model (OR = 0.71, *p* = 0.034, 95% CI = 0.511–0.974]. Conclusions: In a secondary analysis of the national health and nutrition survey data from the US, adherence to the Mediterranean diet was significantly associated with better physical functional abilities among older people.	+2: Med
**Physical Performance + Muscle Strength**							
Talegawkar, 2012 [41] *	InCHIANTI (Invecchiare in Chianti), study, Tuscany, Italy	690 older men and women (people ≥ 65 years) Mean ± SD age: at baseline 73.0 ± 6.24 years	LS	MS: grip strength. PP: walking speed (time to walk 15 feet).	FFQ created for EPIC, validated in this population. Dietary indices: Adherence to a Mediterranean dietary pattern was assessed using the MDS by Trichopoulou et al. [50].	MS: No association was observed for grip strength.	+2: Med
						PP: After a 6-year follow-up, higher adherence to a MD at baseline was associated with a lower risk of low walking speed (OR = 0.48 (95% CI = 0.27, 0.86)). Conclusions: In community-dwelling older adults, higher adherence to a Mediterranean-style diet was associated with a lower risk of low walking speed.	
León-Muñoz, 2014 [42] *	ENRICA cohort, Spain	1815 community-dwelling people aged ≥ 60 years Mean ± SD age: at baseline 68.5 ± 0.3 years	LS	MS: measured with a Jamar dynamometer on the dominant hand. PP: walking speed was assessed using the 3-metre walking speed test.	Validated computerized diet history. Dietary indices: Adherence to a Mediterranean dietary pattern.		+4: Low
					(1) Mediterranean Diet Adherence Screener (MEDAS).	MS: No significant association was observed.	
						PP: Being in the highest tertile of the MEDAS score (highest MD adherence) was associated with reduced risk of slow walking (OR = 0.53; 95% CI = 0.35–0.79).	
					(2) MDS.	MS: Participants in the highest tertile of the MDS had lower risk of low grip strength, but the association was not statistically significant.	
						PP: No association was observed. Conclusions: Among community-dwelling older adults a higher adherence to the MD was associated with reduced risk of slow walking.	
León-Muñoz, 2015 [28]	ENRICA cohort, Spain	1872 community-dwelling people aged ≥ 60 years Mean ± SD age: at baseline: 68.7 ± 0.3 years	LS	MS: strength on the dominant hand was measured with a Jamar dynamometer. PP: walking speed was assessed with the 3-m walking speed test.	Validated computerized diet history. Data-driven: Factor analysis. Two patterns were identified.		+5: Low
					(1) The first was called the “prudent” pattern due to the high consumption of olive oil, vegetables, potatoes, legumes, blue fish, pasta, and meat.	MS: No association was observed	
						PP: A greater adherence to the prudent pattern showed a non-statistically significant tendency to a lower risk of slow walking speed.	
					(2) The second was called the “Westernized” pattern because of the high consumption of refined bread, whole dairy products, and red and processed meat, as well as the low intake of whole grains, fruit, low-fat dairy, and vegetables.	MS: No association was observed.	
						PP: The westernized pattern showed an association with an increasing risk of slow walking speed. Specifically, the OR (95% CI) of slow walking speed across tertiles of the WP were 1, 1.15 (0.74–1.76), and 1.85 (1.19–2.87); *p*-trend = 0.007. Conclusions: Greater adherence to the “Westernized” pattern was associated with increased risk of slow walking speed.	
Granic, 2016 [32]	The Newcastle 85+ Study, UK	791 men and women (living either at home or in a care facility) were followed-up for change in hand grip strength (HGS) and Timed Up-and Go (TUG) test over 5 years. Participants with DP and HGS data 5 years later (*n* = 291). Participants with DP and TUG data 5 years later (*n* = 271) Mean age: the Newcastle 85+ Study targeted 85 years old at baseline	LS	MS: hand grip strength (HGS) was assessed using a hand-held dynamometer. PP: assessed by the Timed Up-and-Go (TUG) test.	24-h multiple-pass dietary recall on two different days of the week, at least one week apart. Data-driven: Cluster analysis. Three dietary patterns (DP) were identified.		+3: Med
					(1) DP1 (“High Red Meat”).	MS: Men in DP1 (“High Red Meat”) had worse overall HGS (β = −1.70, *p* = 0.05) compared with DP2 (“Low Meat”). No association between DP and HGS was observed in women.	
						PP: Men in DP1 and women in DP3 had overall slower TUG than those in DP2 (β = 0.08, *p* = 0.001 and β = 0.06, *p* = 0.01, respectively), but similar rate of decline.	
					(2) DP2 (“Low Meat”).	MS: See above and below.	
						PP: See above.	
					(3) DP3 (“High Butter”).	MS: Men in DP3 (“High Butter”) had a steeper decline in HGS (β = −0.63, *p* = 0.05) than men in DP2 (“Low Meat”). No association between DP and HGS was observed in women.	
						PP: See above. Conclusions: DP high in red meats, potato and gravy (DP1), or butter (DP3) may adversely affect muscle strength and physical performance in later life.	
Xu, 2012 [43] *	1999–2002 NHANES, USA	The final sample size was 2132 for gait speed and 1392 for knee extensor power. Men and women aged 60 years or older Mean ± SD age: 70.4 ± 0.3 (SE) years	CS	MS: knee extensor power. Right knee extensor force production was measured using an isokinetic dynamometer. PP: gait speed; the timed 20-feet walk test was performed at the participant’s usual pace.	24-h multiple-pass dietary recall interview. Dietary indices: The Healthy Eating Index-2005 (HEI-2005), a composite score assessing an individual’s adherence to the 2005 Dietary Guidelines for Americans, was used to measure an individual’s overall diet quality.	MS: Total HEI-2005 scores were positively associated with knee extensor power (*p* for trend = 0.05). Those with HEI-2005 scores in Quartile 4 had a greater knee extensor power compared with those with HEI-2005 scores in the lowest quartile (*p* = 0.04). The associations were no longer statistically significant after further adjustment for PA.	+4: Low
						PP: Total HEI-2005 scores were positively associated with gait speed (*p* for trend = 0.02). Older adults with higher HEI-2005 scores had a faster gait speed (*p* = 0.03 for both Quartile 3 and Quartile 4 versus Quartile 1) compared with those with HEI-2005 scores in the lowest quartile. The associations were no longer statistically significant after further adjustment for PA. Conclusions: Adherence to overall dietary recommendations was associated with better physical performance among older adults.	
Bollwein, 2013 [44]	Region of Nürnberg, Germany	192 community-dwelling older men and women, aged 75 years and older Mean ± SD age: 83 ± 4 years	CS	MS: grip strength was measured with a dynamometer. PP: walking speed.	Administered FFQ of the German part of the EPIC study. Dietary indices: Adherence to a Mediterranean dietary pattern was assessed using the MD score used was the alternate MED score of Fung et al. [51] who adapted the original score of Trichopoulou et al. [50] for a non-Mediterranean population.	MS: No association was observed.	+4: Low
						PP: There was a significant inverse association between “low walking speed” and the MED score; there was an association between a high diet quality and a lower risk of low walking speed. Compared with the lowest quartile (least healthy diet), the participants in the highest quartile (most healthy diet) had a significantly decreased risk of low walking speed (OR (95% CI) = 0.29 (0.09–1.00), *p* for trend = 0.043). Conclusions: There was an association between adherence to a healthy dietary pattern and low walking speed.	
Fougère, 2016 [45]	TRELONG study, Northeast Italy	304 men and women, over 70 years of age at baseline (aged 77 years and over). Mean ± SD age: 86.3 ± 6.8 years	CS	MS: hand grip strength was measured using a dynamometer with the stronger hand. PP: The SPPB was used: standing balance, walking, and chair stand tests. Standing balance tests included tandem, semi tandem and side-by-side stands.	Unclear how dietary data were collected. Dietary indices: Adherence to a Mediterranean dietary pattern was assessed using the Mediterranean Style Dietary Pattern Score (MSDPS).	MS: No correlation was found for hand grip strength.	+1: Med
						PP: A statistically significant association (Regression coefficient = 1.0006; Std. Error = 0.4780; *p*-value = 0.0363) between participants with the highest adherence to the Mediterranean diet (fourth quartile) and high physical performance (SPPB > 7) was found. Conclusions: A statistically significant association between a high adherence to the Mediterranean diet and higher physical performance (SPPB) was found. These findings suggest that MD does not improve the muscle (hand grip) but rather improve global function (indicated by SPPB).	
**Physical Performance + Muscle Strength + Muscle Mass**							
Yokoyama, 2017 [46]	Kusatsu Longitudinal Study, and the Hatoyama Cohort Study, Japan	Community-dwelling Japanese aged 65 years or older. Grip strength: *n* = 781; Gait speed: *n* = 772; Body composition: *n* = 542 Mean ± SD age: according to DVS categories: Lowest: 71.1 ± 4.9; Middle: 71.8 ± 5.1; Highest: 72.8 ± 5.6	LS	MM: Body composition was measured using the InBody 720 device. In this study, lean body mass refers to bone-free lean mass. The sum of non-fat, non-bone tissue in both arms and legs was used to represent ALM. MS: grip strength was measured using hand dynamometers, with the dominant hand.PP: usual gait speed was measured over a straight 11-m walkway.	Participants were asked about consumption frequencies during 1 week for 10 food items. Dietary indices: Dietary variety was assessed at the baseline survey using the DVS (a method of assessing dietary quality based on a count of the number of foods consumed). DVS was categorized into 3 groups.	MM: Dietary variety was not significantly associated with changes in lean body mass or ALM. However, the OR for decline in ALM tended to decrease with increasing DVS at baseline; the multivariable-adjusted OR for decline in ALM was 0.28 (0.07–1.07) for the highest DVS category as compared with the lowest DVS category (*p* for trend = 0.068).	+3: Med
						MS: ORs for decline in grip strength was 0.43 (95% CI = 0.19–0.99), for the highest category of dietary variety score as compared with the lowest category.	
						PP: ORs for decline in usual gait speed was 0.43 (confidence interval, 0.19–0.99), respectively, for the highest category of dietary variety score as compared with the lowest category. Conclusions: Greater dietary variety was associated with lower risk for future declines in physical function (muscle strength and gait speed), but the associations with lean body mass and ALM were less clear. The findings indicate that among older adults, greater dietary variety may help maintain physical performance and muscle strength but not lean mass.	
**Physical Performance + Muscle Mass**							
Smee, 2015 [47]	Canberra, Australia	171 cognitively unimpaired, community-dwelling men and women aged 60 years and over Mean ± SD age: 68.12 ± 6.21 years	CS	MM: Lean mass was assessed by DXA. PP: measured using the SPPB.	Valid semi-quantitative self-administered questionnaire. Dietary indices: Diet quality was determined using two measures.		−4: High
					(1) Healthy Eating Index (HEI)	MM: Lean mass was not significantly associated with the HEI-total score.	
						PP: SPPB was not significantly associated with the HEI-total score.	
					(2) Healthy Diet Indicator (HDI)	MM: In women, there was a weak positive association between HDI score and % lean mass (*r* = 0.20, *p* = 0.03). When controlling for age, there was a weak negative correlation (*r* = 1.19, *p* = 0.03).	
						PP: Men showed weak positive associations between HDI score and SPPB (*r* = 0.26, *p* = 0.04). Conclusions: In women, there was a weak association between better diet quality and higher lean mass, which disappeared when controlling for age. In men there was a weak association between better diet quality and better physical function (SPPB).	
**Sarcopenia**							
Chan, 2016 [29]	Hong Kong, China	Chinese community-dwelling men and women, aged 65 years or older. The final sample size for the cross-sectional analyses was 3957 and for the prospective analyses was 2948 Mean ± SD age: At baseline: Non-sarcopenic participants (*n* = 3667): 72.2 ± 5.0 years; Sarcopenic participants (*n* = 290): 76.2 ± 6.1 years	LS	Sarcopenia was defined according to the AWGS algorithm. An individual with low muscle mass, low muscle strength, and/or low physical performance was categorized as having sarcopenia.	Validated semi-quantitative FFQ Data-driven: factor analysis.		+5: Low
					(1) “Vegetables-fruits” dietary pattern.	At baseline, men with higher “vegetables-fruits” dietary pattern score, and higher “snacks-drinks-milk products” dietary pattern score had lower likelihood of being sarcopenic. Men in the highest quartile of “vegetables-fruits” pattern score (adjusted OR = 0.60, 95% CI = 0.36–0.99, *p* for trend = 0.034) showed reduced likelihood of sarcopenia compared with men in the lowest quartile. No such associations were observed in women. No significant associations were found between any of the dietary patterns and 4-year incident sarcopenia.	
					(2) “Snacks-drinks-milk products” dietary pattern.	See above. Higher quartile of “snacks-drinks-milk products” pattern score was associated with lower likelihood of sarcopenia in men (adjusted OR = 0.41, 95% CI = 0.24–0.70, *p* for trend < 0.001). No such associations were observed in women.	
					(3) “meat-fish” dietary pattern.	Sarcopenia: No association was observed.	
					Dietary indices.		
					(1) Adherence to a Mediterranean dietary pattern was assessed using the MDS by Trichopoulou et al. [50]	Sarcopenia: No associations were found between MDS and sarcopenia.	
					(2) The Diet Quality Index-International (DQI-I) was calculated.	Sarcopenia: At baseline, men with higher DQI-I score had lower likelihood of being sarcopenic. Men in the highest quartile of DQI-I had reduced likelihood of sarcopenia (adjusted OR = 0.50, 95% CI = 0.31–0.81, *p* for trend = 0.004) compared with men in the lowest quartile. No such associations were observed in women. No significant associations were found between dietary patterns and 4-year incident sarcopenia. Conclusions: Higher DQI-I score, higher “vegetables-fruits” dietary pattern score, and higher “snacks-drinks-milk products” dietary pattern score were associated with lower likelihood of prevalent sarcopenia in Chinese older men.	
Hashemi, 2015 [30]	Tehran, Iran	300 elderly men and women (55 years old and older) who lived in the sixth district of Tehran Mean ± SD age: 66.8 ± 7.72 years	CS	Sarcopenia was defined according to EWGSOP criteria, based on a combination of relatively low appendicular muscle mass with either low muscle strength or low muscle performance.	Administered FFQ. Data-driven: PCA. Three major dietary patterns were identified.		+2: Med
					(1) Mediterranean, defined as a dietary pattern with high factor loadings (>0.4) in food groups such as olives and olive oil, low and high carotenoid vegetables, tomatoes, whole grains, nuts, fish, fresh and dried fruits, and pickles.	Participants in the highest tertile of the MD pattern had a lower odds ratio for sarcopenia than those in the lowest tertile (OR = 0.42; 95% CI = 0.18–0.97; *p* for trend = 0.04).	
					(2) Western, defined as a dietary pattern with high factor loadings in tea, soy, sweets, desserts, sugars, and fast foods.	Adherence to the Western dietary pattern was not associated with sarcopenia (OR = 0.51; 95% CI = 0.21–1.24; *p* for trend = 0.13).	
					(3) Mixed, identified as a pattern with high factor loadings in the following food groups: animal proteins, legumes, potatoes, and refined grains.	Adherence to the Mixed dietary pattern did not affect the odds of sarcopenia (OR = 1.45; 95% CI = 0.66–3.19; *p* for trend = 0.95). Conclusions: The study suggests that adherence to the MD is associated with lower odds of sarcopenia among Iranian older adults.	

DQ: diet quality; FICSIT, Frailty and Injuries: Cooperative Studies of Intervention Techniques; SD: standard deviation; KNHANES: Korean National Health Examination and Nutrition Survey; CS: Cross-sectional study; LS: Longitudinal study; DXA: dual-energy X-ray absorptiometry; ALM: Appendicular lean mass; EPIC: European Prospective Investigation into Cancer and Nutrition; FFQ: food frequency questionnaire; PA: physical activity; DVS: Dietary Variety Score; PCA: Principal component analysis; SPPB: short physical performance battery; HCS: Hertfordshire Cohort Study; MDS: Mediterranean diet score; MD: Mediterranean diet; NHANES: National Health and Nutrition Examination Survey; AWGS: Asian Working Group for Sarcopenia; EWGSOP: European Working Group on Sarcopenia in Older People. *^a^* Risk of bias quality rating: total score −9 to −3 = high risk of bias; −2 to +3 = medium risk of bias; +4 to +10 = low risk of bias. *^b^* The Korean diet typically consists of white rice, soup and side dishes with many plant foods, and is characterized as a low-fat and high-vegetable diet. *^c^* A “prudent” dietary pattern reflects recommendations for a healthy diet. A “prudent” diet score was calculated for each participant that indicates compliance with the pattern. A high prudent diet score (in the upper part of distribution of scores) indicates a diet characterised by high consumption of fruit, vegetables, whole-grain cereals and oily fish, but low consumption of white bread, chips, sugar and full fat dairy products. *^d^* The Senior Fitness Test (SFT) battery consisted of five measurements of physical fitness: (1) number of chair stands during 30 s to assess lower-body strength, (2) arm curl to assess upper-body strength, (3) chair sit and reach to assess lower-body (hamstring) flexibility, (4) back scratch to assess upper-body (shoulder) flexibility and (5) 6-min walk test to measure aerobic endurance (distance walked in 6 min). *^e^* MABAT ZAHAV: Physical function data were collected using subjective criteria and therefore not relevant to this review. * There is another included study related to this cohort or survey in this table (these studies are separate studies within the same cohort).

## Supplementary Materials

The following are available online at http://www.mdpi.com/2072-6643/10/3/308/s1, Table S1: Search strategy and terms (application to the Ovid MEDLINE database); Table S2: Quality assessment form used to assess risk of bias of studies included in the review, alongside guidance notes relating to the scoring system; Table S3: Summary of scoring results in terms of risk of bias (low, medium or high) of all studies included in the review.
