# Supplementary material for: Diet Quality and Sarcopenia in Older Adults: A Systematic Review
Source: Nutrients. 2018 Mar 5;10(3):308. doi: 10.3390/nu10030308 (PMC5872726; doi:10.3390/nu10030308)
Supplement: Supplementary file 1 [file nutrients-10-00308-s001.zip › nutrients-272424-supplementary Table S1 _27 02 2018.doc]

**Table S1:** Search strategy and terms (application to the Ovid MEDLINE database).

| **Database** | **Search terms** | **Results** |
| --- | --- | --- |
| Ovid MEDLINE(R) without Revisions 1996 to July week 4 2016  **Limits:**  English  Human  9/8/16 | 1 Sarcopenia/ 1635  2 Muscular Atrophy/ 9709  3 sarcopenia.ti,ab. 2551  4 muscle*.ti,ab. 561106  5 exp Body Composition/ 43690  6 muscle fatigue/ or muscle strength/ or hand strength/ or pinch strength/ or muscle tonus/ 35541  7 motor activity/ or physical exertion/ or physical endurance/ or physical fitness/ or postural balance/ or psychomotor performance/ 241752  8 physical function*.ti,ab. 14786  9 Walking/ 24975  10 frail*.ti,ab. 10604  11 gait/ 21422  12 gait.ti,ab. 29974  13 "Activities of Daily Living"/ 55516  14 quality of life/ 141515  15 exp Posture/ 66127  16 psychomotor performance.ti,ab. 1460  17 posture.ti,ab. 20827  18 "timed get up and go".ti,ab. 113  19 bioimpedence.ti,ab. 27  20 bio impedence.ti,ab. 3  21 DXA.ti,ab. 7191  22 (lean adj2 mass).ti,ab. 9217  23 (fat adj2 mass).ti,ab. 17827  24 1 or 2 or 3 or 4 or 5 or 6 or 7 or 8 or 9 or 10 or 11 or 12 or 13 or 14 or 15 or 16 or 17 or 18 or 19 or 20 or 21 or 22 or 23 1117902  25 Food Habits/ 25636  26 exp Diet/ 230626  27 exp Food/ 1097754  28 (diet or diets).ti,ab. 257820  29 healthy eating.ti,ab. 3509  30 healthful eating.ti,ab. 312  31 eating habit*.ti,ab. 3369  32 eating pattern*.ti,ab. 1746  33 food habit*.ti,ab. 1559  34 dietary.hw. 176321  35 25 or 26 or 27 or 28 or 29 or 30 or 31 or 32 or 33 or 34 1360137  36 24 and 35 78733  37 aged/ 2584483  38 exp Aged/ 2614395  39 senior*.ti,ab. 26814  40 old age.ti,ab. 19987  41 older.ti,ab. 286381  42 37 or 38 or 39 or 40 or 41 2762185  43 36 and 42 9576  44 limit 43 to english language 8909  **45 limit 44 to humans 8608** | 8608 references downloaded |
