# Supplementary material for: Diet Quality and Sarcopenia in Older Adults: A Systematic Review
Source: Nutrients. 2018 Mar 5;10(3):308. doi: 10.3390/nu10030308 (PMC5872726; doi:10.3390/nu10030308)
Supplement: Supplementary file 1 [file nutrients-10-00308-s001.zip › nutrients-272424-supplementary Table S2 _27 02 2018.doc]

**Table S2:** Quality assessment form used to assess risk of bias of studies included in the review, alongside guidance notes relating to the scoring system.

| **Quality assessment** | | | **Scoring system**  For each field, the guidance notes below were used to give a brief response to each question, together with a suitable quality score or risk of bias score:  **High (-1), Medium (0) or Low (+1)** |
| --- | --- | --- | --- |
| Criterion | Score | Comment |  |
| 1. Study design appropriate? |  |  | Score:  -1: if design and/or setting are ambiguously described, or obviously bias inducing or unsuitable for the objectives and stated conclusions  0: if design not obviously inappropriate; setting is possibly restricting but reflected in the scope of the objectives and the conclusions drawn (e.g. hospital settings)  +1: if design appropriate for the outcome and setting chosen to minimise bias |
| 2. Adequate description of study participants? |  |  | Score:  -1: Little or no information given  0: The inclusion/exclusion criteria defining the study subjects or characteristics of the subjects are given in some way; at least one of physical activity and SES (socioeconomic status) given along with at least one other useful measure such as age, gender, ethnicity, co-morbidities, or smoking status  +1: The inclusion/exclusion criteria defining the study subjects and characteristics of the subjects are clearly given; at least one of physical activity and SES given along with at least two other useful measures such as age, gender, ethnicity, co-morbidities, or smoking status |
| 3. Measurements of diet quality reliably ascertained? |  |  | Score:  -1: Inadequately explained or obviously unsuitable  0: Adequate description and reliability/suitability of at least one of the following: instruments, technique/definition/protocol, people, place  +1: Detailed description and reliability of one and at least adequate description of the others |
| 4. Measurements of outcomes reliably ascertained? |  |  | Score:  -1: Inadequately explained or obviously unsuitable  0: Adequate description of at least two of instruments, technique/definition/protocol, people, place, reliability/suitability  +1: Detailed description and reliability of one and at least adequate description of the others |
| 5 Consideration for the effects of important confounding factors? |  |  | Score:  -1: One important confounding factor (ICF)^[1]^ controlled for in tables (tables that directly associate diet quality with an outcome of interest), nothing for the others (regardless of whether they were measured or not)  0: Most ICFs controlled for in tables, or 3 – 4 are adjusted for in regression  +1: 5 or more ICFs adjusted for in regression  N.B. If most of the adjusted-for ICFs are in a separate analysis to determine their effect/significance, rather than being directly adjusted for, give a score of 0 rather than +1. |
| 6. Outcome assessment blind to diet quality status? |  |  | Score:  Some details given: +1  Otherwise: 0 |
| 7. What proportion of the target population was followed up? |  |  | Score:  -1: if % FU is not given, unclear, or very low (below 70%)  0: if % FU is low to average (70-90%)  +1: if % FU is high (over 90%) |
| 8. Info on non-participants |  |  | Score:  -1: if no information, or very little, about either exclusions or losses from the original cohort or comparisons between participants and non-participants is given; or if whatever information is given suggests a high potential for bias  0: if some information is given on both counts, or if one is given completely and the other not at all; or if complete information is given but suggests a medium potential for bias  +1: if one is given completely and the other has at least some information, and the information given has little or no potential for bias |
| 9. Analysis rigorous and appropriate? |  |  | Score:  -1: if no statistical analyses are carried out (just tables or description)  0: if tables of means and differences are given with statistical tests (e.g. t-tests), or some regression but without clear/valid measure of association  +1: if regression (or similar technique) is used which gives a valid measure of association (e.g. odds ratios, hazard ratios, relative risks) |
| 10. Sample size |  |  | Score:  -1: if extremely ambiguous, not given, or small (under 100)  0: if average (100 to 1000)  +1: if large (over 1000) |
| **Overall quality rating** (sum of scores): |  |  | Total score can range from -9 to +10. The overall risk of bias in relation to the review question was categorised as follows:  -9 to -3 = high risk of bias  -2 to +3 = medium risk of bias  +4 to +10 = low risk of bias |

1. Important Confounding Factors for this review: Age, Gender, Physical activity, Ethnicity, Socioeconomic status/education, Co-morbidities, Smoking status.
