# Supplementary material for: Diet Quality and Sarcopenia in Older Adults: A Systematic Review
Source: Nutrients. 2018 Mar 5;10(3):308. doi: 10.3390/nu10030308 (PMC5872726; doi:10.3390/nu10030308)
Supplement: Supplementary file 1 [file nutrients-10-00308-s001.zip › nutrients-272424-supplementary Table S3 _27 02 2018.doc]

**Table S3:** Summary of scoring results in terms of risk of bias (low, medium or high) of all studies included in the review. (Total score -9 to -3 = high risk of bias; -2 to +3 = medium risk of bias; +4 to +10 = low risk of bias).

| *First author, year* | *Question and risk of bias* | | | | | | | | | | |
| --- | --- | --- | --- | --- | --- | --- | --- | --- | --- | --- | --- |
|  | **1. Study design** | **2. Study participants** | **3. Measurements of diet quality** | **4. Measurements of outcomes** | **5. Confounding factors** | **6. Blinding** | **7. % Follow-up** | **8. Info on non-participants** | **9.** **Analysis** | **10. Sample size** | **Overall quality rating: Risk of bias** |
| Akbaraly, 2013 | +1 | +1 | 0 | -1 | -1 | 0 | 0 | 0 | +1 | +1 | +2: Med |
| Bernstein, 2002 | 0 | -1 | +1 | -1 | -1 | 0 | -1 | -1 | +1 | -1 | -4: High |
| Bollwein, 2013 | 0 | +1 | +1 | 0 | 0 | 0 | +1 | 0 | +1 | 0 | +4: Low |
| Chan, 2016 | +1 | +1 | 0 | 0 | +1 | 0 | 0 | 0 | +1 | +1 | +5: Low |
| Fougère, 2016 | 0 | +1 | -1 | 0 | +1 | 0 | -1 | 0 | +1 | 0 | +1: Med |
| Granic, 2016 | +1 | -1 | +1 | +1 | +1 | 0 | -1 | 0 | +1 | 0 | +3: Med |
| Hashemi, 2015 | 0 | +1 | +1 | 0 | +1 | 0 | -1 | -1 | +1 | 0 | +2: Med |
| Kojima, 2015 | +1 | +1 | 0 | 0 | -1 | 0 | -1 | 0 | +1 | 0 | +1: Med |
| León-Muñoz, 2014 | +1 | 0 | 0 | 0 | +1 | 0 | 0 | 0 | +1 | +1 | +4: Low |
| León-Muñoz, 2015 | +1 | +1 | 0 | 0 | +1 | 0 | 0 | 0 | +1 | +1 | +5: Low |
| Martin, 2011 | +1 | +1 | +1 | 0 | 0 | 0 | 0 | -1 | +1 | 0 | +3: Med |
| Milaneschi, 2011 | +1 | +1 | 0 | 0 | 0 | 0 | -1 | 0 | +1 | 0 | +2: Med |
| Nikolov, 2016 | +1 | +1 | 0 | 0 | +1 | 0 | -1 | -1 | +1 | +1 | +3: Med |
| Oh, 2014 | +1 | +1 | 0 | 0 | +1 | 0 | -1 | -1 | +1 | +1 | +3: Med |
| Perälä, 2016 | +1 | +1 | +1 | +1 | 0 | 0 | -1 | 0 | +1 | +1 | +5: Low |
| Rahi, 2014 | +1 | +1 | +1 | 0 | +1 | 0 | -1 | 0 | -1 | 0 | +2: Med |
| Robinson, 2008 | +1 | +1 | +1 | 0 | -1 | 0 | +1 | 0 | +1 | +1 | +5: Low |
| Shahar, 2012 | +1 | +1 | 0 | 0 | +1 | 0 | -1 | 0 | +1 | +1 | +4: Low |
| Smee, 2015 | -1 | 0 | 0 | -1 | -1 | 0 | -1 | -1 | +1 | 0 | -4: High |
| Talegawkar, 2012 | +1 | +1 | 0 | -1 | +1 | 0 | -1 | 0 | +1 | 0 | +2: Med |
| Xu, 2012 | +1 | +1 | 0 | 0 | +1 | 0 | -1 | 0 | +1 | +1 | +4: Low |
| Yokoyama, 2017 | +1 | +1 | 0 | 0 | +1 | 0 | -1 | 0 | +1 | 0 | +3: Med |
| Zbeida, 2014 | +1 | +1 | 0 | -1 | +1 | 0 | -1 | -1 | +1 | +1 | +2: Med |
